# HIF1α is not a target of 14q deletion in clear cell renal cancer

**DOI:** 10.1038/s41598-020-74631-7

**Published:** 2020-10-19

**Authors:** Niraj Shenoy

**Affiliations:** 1 Department of Medicine (Oncology), Albert Einstein College of Medicine, Montefiore Medical Center, New York, 10461 USA; 2 Experimental Therapeutics Program, Albert Einstein Cancer Center, Albert Einstein College of Medicine, New York, 10461 USA

**Keywords:** Cancer, Computational biology and bioinformatics, Genetics, Oncology

## Abstract

HIF1α has been termed a tumor-suppressor in clear cell renal cell carcinoma (ccRCC), primarily based on functional proliferation studies in cell lines (in vitro and in vivo) with genetic manipulation, and the adverse prognosis of 14q-deleted ccRCC patients. In other malignancies, however, HIF1α has an established tumor-promoting role. Therefore, this study sought to further examine the role of HIF1α in ccRCC using bioinformatic analyses of 530 ccRCC patients from The Cancer Genome Atlas (TCGA) and The Cancer Proteome Atlas (TCPA) registries. Although lower copy numbers of *HIF1A* (encoding HIF1α, located at 14q23.2) was associated with worse survival, there was no survival difference based on either *HIF1A* mRNA or HIF1α protein expression. Interestingly, *L2HGDH* (L-2-Hydroxyglutarate Dehydrogenase), a recently characterized epigenetic modulating ccRCC tumor-suppressor with a marked impact on survival, was found to be located only ~ 11.5Mbp from *HIF1A* on 14q (at 14q21.3). *L2HGDH* was therefore co-deleted in ~ 95% of 14q deletions involving *HIF1A* locus. Remarkably, *HIF1A* CNV had a markedly stronger correlation with *L2HGDH* expression (Rho = 0.55) than its own gene expression (Rho = 0.27), indicating high preserved-allele compensation of *HIF1A*. Genetic loss of *HIF1A* was therefore associated with a much greater reduction of *L2HGDH* gene expression than its own gene expression, providing a possible explanation for survival differences based on *HIF1A* CNV and mRNA expression. Furthermore, in 14q-deleted ccRCC patients with complete (uncensored) survival data, in the relatively rare cases where genetic loss of *HIF1A* occurred without genetic loss of *L2HGDH* (n = 5), the survival was significantly greater than where there was simultaneous genetic loss of both (n = 87) (mean survival 1670.8 ± 183.5 days vs 885.1 ± 78.4 days; p = 0.007). In addition, there was no correlation between *HIF1A* mRNA and HIF1α protein expression in ccRCC (R = 0.02), reflecting the primarily post-translational regulation of HIF1α. Lastly, even between *L2HGDH* and *HIF1A* loci, 14q was found to have several other yet-to-be-characterized potential ccRCC tumor-suppressors. Taken together, the data indicate that HIF1α is not a target of 14q deletion in ccRCC and that it is not a tumor-suppressor in this malignancy.

## Introduction

Since its discovery^1^, purification and early characterization^2–6^, Hypoxia-inducible factor 1 (HIF1) has been of immense interest to cancer biologists^7–9^. It is a heterodimeric protein, composed of HIF1α (encoded by *HIF1A*, gene locus at 14q23.2) and HIF1β (encoded by ARNT, gene locus at 1q21.3). HIF1α is primarily regulated by O2-dependent prolyl hydroxylation and subsequent ubiquitilation by Von-Hippel-Lindau (VHL)-containing E3-ubiquitin ligase complex, followed by proteasomal degradation^10–14^. HIF1 functions as a transcription factor, enhancing the expression of genes involved in angiogenesis, metabolic adaptation, proliferation, invasion, resistance, and survival^9,15^. HIF1α is commonly overexpressed in cancers, both as a result of intra-tumoral hypoxia and O2-independent upregulation by oncogenic mutations and pathways^16–18^. HIF1α overexpression has been associated with poor prognosis in several cancers (non-small cell lung, breast, head and neck, esophageal, pancreatic, cervical, ovarian, endometrial, GI stromal)^9,15^, providing strong evidence for a tumor-promoting role in these cancers.

In clear cell renal cell carcinoma (ccRCC), however, the role of HIF1α has been debated. ccRCC accounts for ~ 80% of all kidney cancer, and VHL inactivation, either through genetic sequence alteration or promoter methylation occurs in ~ 86% of all ccRCC as an early initiating feature during pathogenesis^19,20^. This results in the constitutive activation of the HIF pathway, which also consists of HIF2α. HIF2α was discovered a few years after HIF1α^21^, has a 48% sequence identity with HIF1α, and also dimerizes with HIF1β primarily upon hypoxic induction to transcribe a hypoxia program. Although there is a varying degree of overlap, there are genes regulated independently by HIF1α or HIF2α, as heterodimers with HIF1β, which can differ significantly with cellular contexts^22^. While HIF2α has been found to have a clear oncogenic role in ccRCC with a HIF2α inhibitor in the drug development pipeline^23–25^, evidence for the role of HIF1α in this disease has been conflicting. The reported protein expression of HIF1α in ccRCC has varied widely, ranging from 17 to 95% positivity^26–29^ and varying association (favorable/unfavorable) with survival^28,30–32^, with one study indicating contrasting prognostic implications with nuclear or cytoplasmic HIF1α^33^. (The wide range of HIF1α ‘positivity’ was due to differing definitions of positivity. Although differing distinctions of high/low HIF1α and institutional variations may have contributed, the exact reasons for the conflicting reports on prognostic implications of HIF1α in ccRCC were unclear.)

HIF1α has been shown to inhibit proliferation of ccRCC cell lines in vitro and in nude xenograft mouse models^34,35^, and has also been shown to inhibit c-myc^29,36–38^. *HIF1A* has been termed a ‘target of 14q deletion’ in ccRCC and a kidney cancer suppressor gene, based on these functional proliferation studies in ccRCC cell lines, the adverse prognosis of 14q-deleted ccRCC patients (large 14q deletions are seen in ~ 40% of ccRCC) and loss of function mutations of *HIF1A* (*HIF1A* mutation occurs in ~ 1% of ccRCC tumors (TCGA)^35^. The adverse prognosis of 14q-deleted ccRCC has been attributed to the loss of *HIF1A*^35,39^. However, conversely, the TRACK (TRAnsgenic Cancer of the Kidney) mouse RCC model, arguably the most representative RCC oncogenesis model to date given the histologic similarities with kidney manifestations of VHL loss, was shown to develop RCC with constitutively active HIF1α expression in the proximal tubules (but not HIF2α)^40,41^. In addition, in the RENCA (syngeneic mouse RCC) model, HIF1α has been shown to promote invasion/migration in vitro^42^. Furthermore, recently, HIF2α inhibitor-resistant ccRCC patient-derived xenograft tumors were observed to have significantly greater *HIF1A* mRNA expression than HIF2α inhibitor-sensitive ccRCC tumors, raising the possibility that HIF1α may be particularly oncogenic in ccRCC tumors with no response despite HIF2α inhibition^43^.

In this study, bioinformatic analysis of a large number of ccRCC patients from The Cancer Genome Atlas (TCGA) dataset and The Cancer Proteome Atlas (TCPA) dataset was performed with the following objectives: one, to study the overall survival impact of *HIF1A*/HIF1α in ccRCC patients; two, to determine the relation of *HIF1A* to a recently characterized ccRCC tumor suppressor gene *L2HGDH*^44,45^; three, to identify other potential 14q tumor suppressors in ccRCC between the *L2HGDH* locus and *HIF1A* locus; and four, to ascertain the implications of the findings in the context of previously published literature on HIF1α in ccRCC.

## Results

### Although lower *HIF1A* CNV was associated with inferior survival in ccRCC, there was no difference in survival based on HIF1A expression, both at mRNA and protein levels

**Figure 1:**
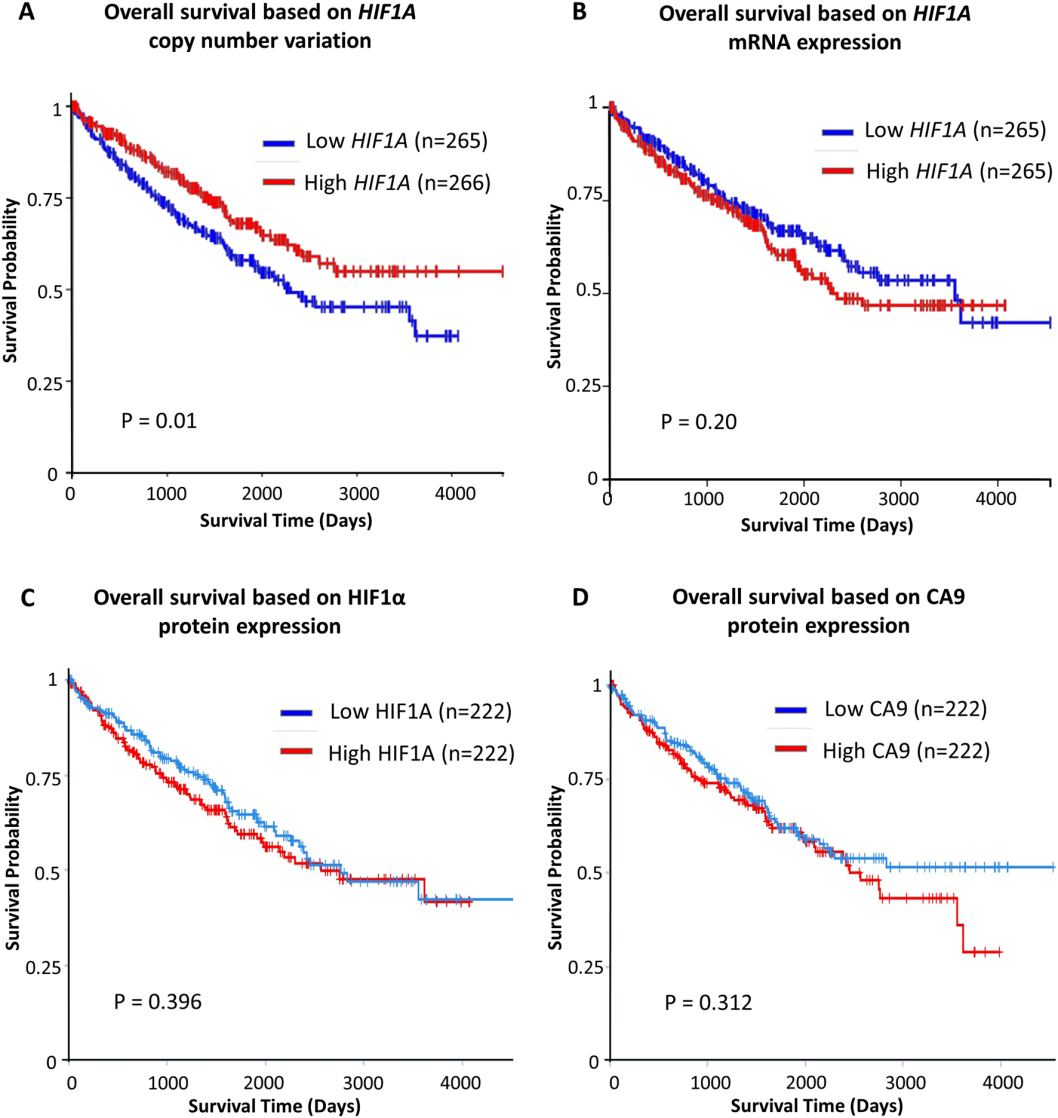
Survival analyses in ccRCC (TCGA) based on *HIF1A* CNV, *HIF1A* mRNA expression, HIF1α protein and CA9 protein expression. (**A**) Lower *HIF1A* CNV (< -0.0297 log2(copy-number/2)) in primary ccRCC tumors was associated with worse overall survival than higher *HIF1A* CNV (≥ -0.0297 log2(copy-number/2)) (log-rank test p = 0.01, n = 531, TCGA clear cell RCC). (**B**) However, there was no significant difference in survival between ccRCC patients with lower *HIF1A* mRNA expression in primary tumors (< 19.4 log2(fpkm-uq + 1)) and higher *HIF1A* mRNA expression (≥ 19.4 log2(fpkm-uq + 1)) (log-rank test p = 0.33, n = 530, TCGA clear cell RCC). Fpkm-uq (Fragments per kilobase of transcript per million mapped reads- upper quartile) is an RNA-sequencing-based expression normalization method. (**C**) Similarly, no significant survival difference between ccRCC patients with lower HIF1α protein expression and higher HIF1α protein expression based on Reverse Phase Protein Array functional proteomics (log-rank test p = 0.4, n = 444, The Cancer Proteome Atlas). (**D**) Also, no significant survival difference between ccRCC patients with lower CA9 protein expression and higher CA9 protein expression (log-rank test p = 0.4, n = 444). In fact, the curves separated beyond ~ Day 2500 and at Day 4000, the higher CA9 expression group had a lower survival rate of 28% compared to that of 51% in the lower CA9 expression group.

Consistent with previous reports of 14q loss, lower *HIF1A* CNV was associated with worse overall survival than higher *HIF1A* CNV in ccRCC (log-rank test p = 0.01, n = 532, Fig. 1A). However, there was no significant difference in survival between ccRCC patients with lower *HIF1A* mRNA expression and higher *HIF1A* mRNA expression (log-rank test p = 0.33, n = 602, Fig. 1B). Likewise, there was no significant difference in survival between ccRCC patients with lower HIF1α protein expression and higher HIF1α protein expression (log-rank test p = 0.4, n = 444, Fig. 1C). Furthermore, Carbonic Anhydrase 9 (CA9) expression, a marker of HIF1 functional activity^46^, also had no significant impact on overall survival (log-rank test p = 0.31, n = 444, Fig. 1D). In fact, at Day 4000, the higher CA9 expression group had a lower survival rate of 28% compared to that of 51% in the lower CA9 expression group.

Taken together, the inferior survival with lower *HIF1A* CNV was not explained by survival based on *HIF1A* mRNA or protein expression or functional activity. The lack of survival difference based on both mRNA and protein expression in this large cohort of ccRCC patients, strongly argue against a tumor suppressive role for HIF1α in ccRCC patients.

### *HIF1A* is co-deleted with *L2HGDH* in a large majority of 14q- deleted ccRCC

**Figure 2:**
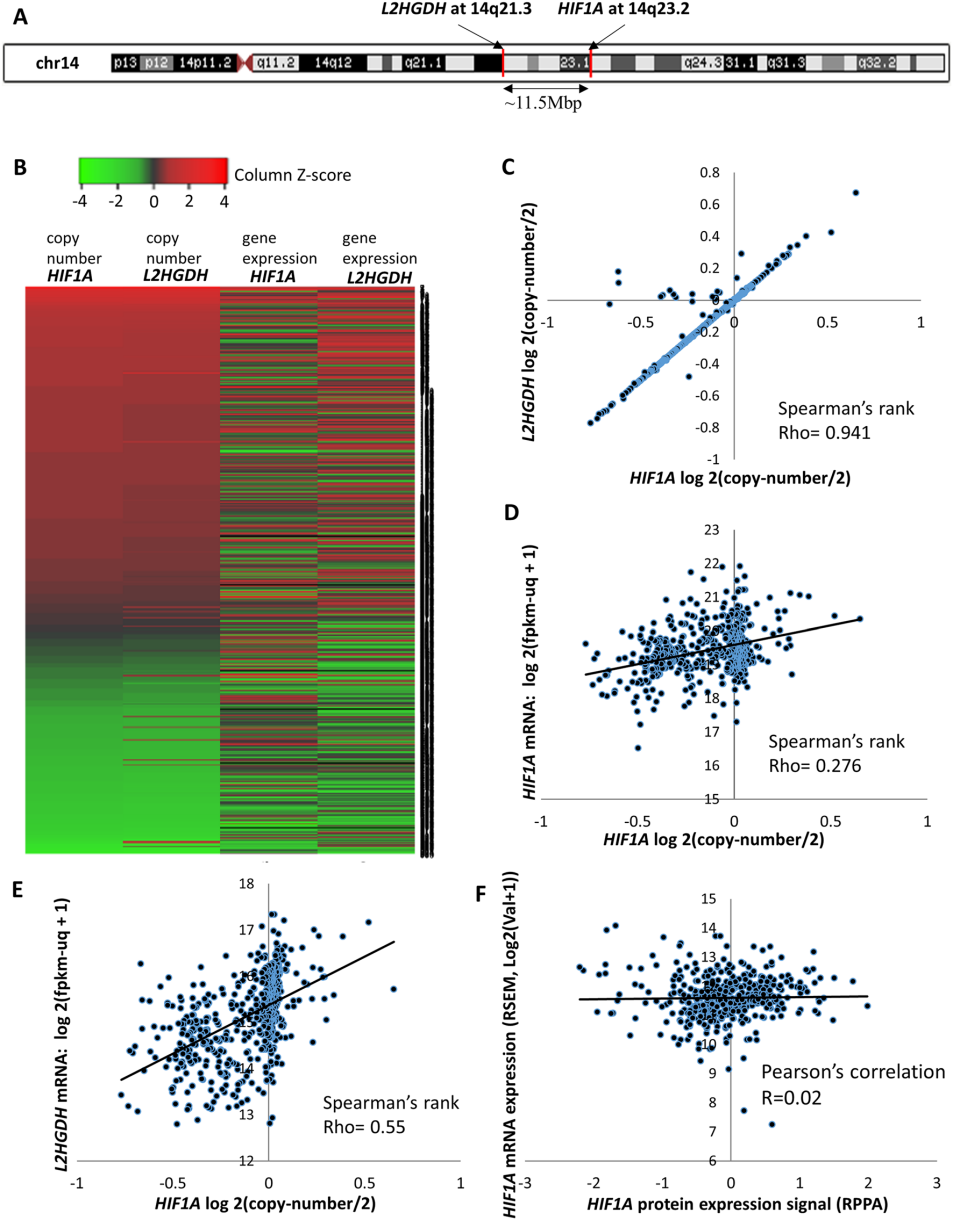
(**A**) Depiction of *L2HGDH,* a recently characterized epigenetic modulating ccRCC tumor suppressor gene with a marked impact on survival, located only ~ 11.5Mbp from *HIF1A* on 14q. (**B**) Heatmap to visualize the relationship between *HIF1A* CNV, *L2HGDH* CNV, *HIF1A* gene expression, *L2HGDH* gene expression in primary ccRCC tumors. The heatmap reveals that there is very strong correlation between *HIF1A* CNV and *L2HGDH* CNV, and that *HIF1A* CNV appears to correlate stronger with *L2HGDH* gene expression rather than its own gene expression. (TCGA clear cell RCC, n = 530). (**C**–**F**) Scatter plots showing the strength of correlation between (**C**) *HIF1A* CNV and *L2HGDH* CNV; (**D**) *HIF1A* CNV and *HIF1A* mRNA expression; (**E**) *HIF1A* CNV and *L2HGDH* mRNA expression; (**F**) *HIF1A* mRNA and HIF1α protein expression; in ccRCC primary tumors (TCGA and TCPA clear cell RCC). Spearman’s rank Rho values and Pearson’s correlation R value are as indicated. The strength of correlation between *HIF1A* CNV and *L2HGDH* mRNA expression was 2-times that between *HIF1A* CNV and *HIF1A* mRNA expression, indicating high preserved allele compensation of *HIF1A*. Furthermore, there was no correlation between *HIF1A* mRNA and HIF1α protein expression.

In order to further investigate the difference in survival between stratification based on CNV versus mRNA expression, the CNV and mRNA expression of *HIF1A* in each ccRCC sample of the TCGA dataset was mapped, along with the CNV and mRNA expression of *L2HGDH* (Fig. 2B), a recently characterized epigenetic modulating ccRCC tumor suppressor gene with a marked impact on survival^44^, which happens to be located only ~ 11.5Mbp from *HIF1A* on 14q (Fig. 2A).

The L2HGDH protein is a flavin adenine dinucleotide (FAD)-dependent enzyme that oxidizes L-2-hydroxyglutarate (L2HG) to alpha-ketoglutarate. The expression of *L2HGDH* is markedly lower in ccRCC compared to normal kidney tissue [TCGA KIRC, ~ 40% reduction in log_2_(TPM + 1) units, Fig. S2C]. Loss of L2HGDH in ccRCC leads to the accumulation of L2HG—an oncometabolite that inhibits the Ten-Eleven-Translocation enzymes, resulting in an adverse loss of hydroxymethylcytosine and gain of methylcytosine. This epigenetic aberrancy mediated by L2HG results in inhibition of expression of multiple tumor suppressor genes in ccRCC^44,45,47^. The accumulation of 2-hydroxyglutarate in ccRCC is 6 times that of control kidney tissue (MSKCC ccRCC metabolomic atlas^48^).

Ninety five percent of 14q deletions that involved the *HIF1A* locus (at 14q23.2) also involved the *L2HGDH* locus (at 14q21.3). *HIF1A* CNV correlated very strongly with that of *L2HGDH* CNV (Spearman’s rank Rho = 0.94, Fig. 2B,C).

### Copy number variation of *HIF1A* has a strong correlation with *L2HGDH* expression, but a weak correlation with its own expression in ccRCC

**Figure 3:**
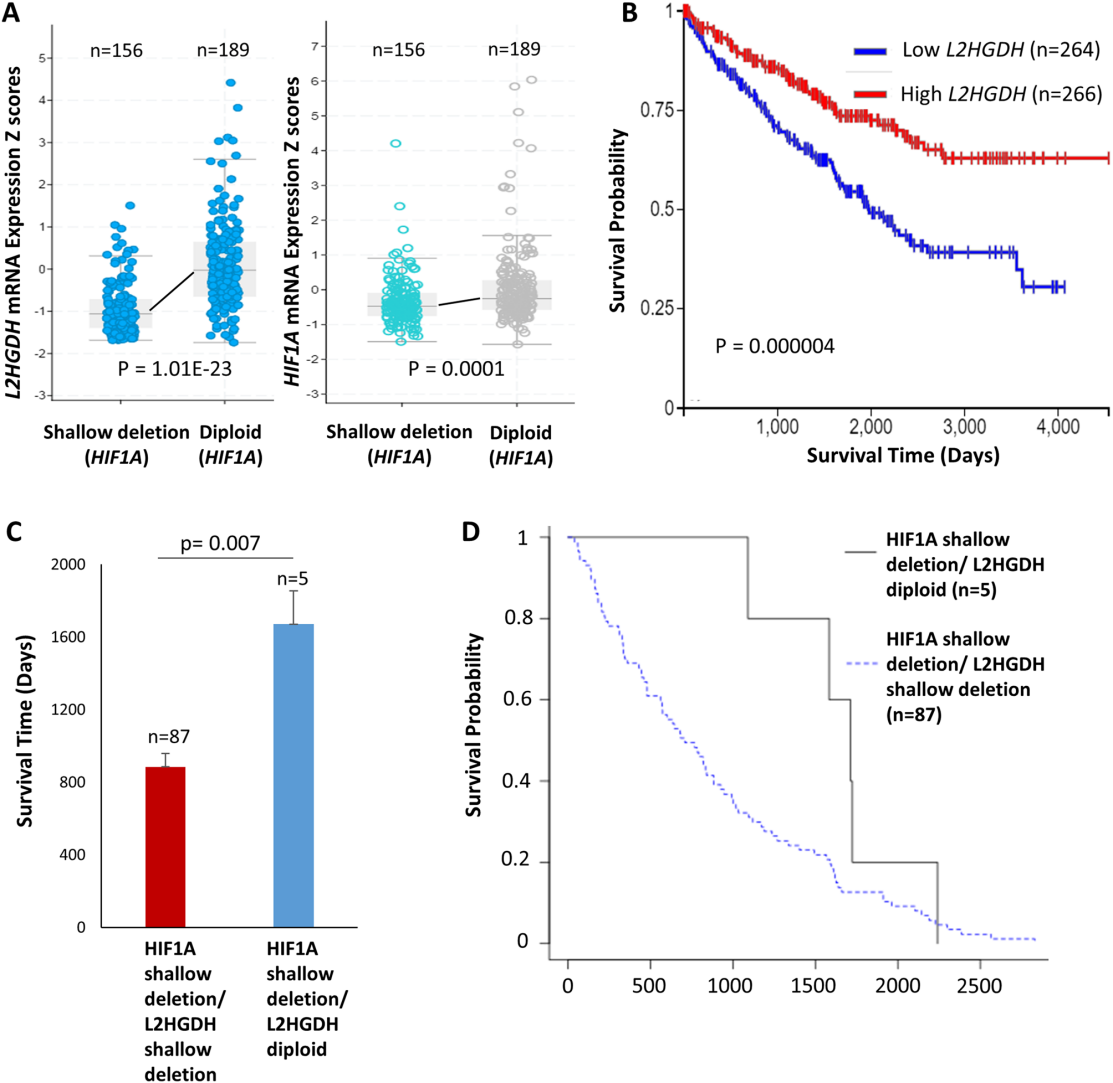
(**A**) Genetic loss (shallow deletions) of *HIF1A* was associated with a much greater reduction of *L2HGDH* expression than its own gene expression (TCGA ccRCC, cbioportal). (**B**) Survival based on *L2HGDH* expression with the same dataset (TCGA ccRCC, n = 530). Higher *L2HGDH* expression (≥ 15.16 log2(fpkm-uq + 1)) was associated with significantly improved survival (log-rank p value as depicted). (**C**) In ccRCC patients with complete (uncensored) survival data (TCGA), patients with “*HIF1A* shallow deletion + *L2HGDH* diploid” had significantly greater mean survival (1670.8 days + /- 183.5 days, n = 5) compared to patients with “*HIF1A* shallow deletion + *L2HGDH* shallow deletion” (885.1 days + /- 74.3 days, n = 87) (t-test p value = 0.007). Data represented as means ± s.e.m. (**D**) Kaplan Meier survival curves of the two groups showing a wide separation between the curves (Peto and Peto modification of the Gehan- Wilcoxon test ‘p’ value = 0.033). Put together, when genetic loss of *HIF1A* occurs without genetic loss of *L2HGDH* in ccRCC*,* the survival is significantly greater than when there is simultaneous genetic loss of *HIF1A* and *L2HGDH*, which argues against *HIF1A* being a target of 14q deletion, and against *HIF1A* being a 14q tumor-suppressor in ccRCC.

Given the strong associations between *L2HGDH* CNV and gene expression, and between *HIF1A* and *L2HGDH* CNV, *HIF1A* CNV had a strong correlation with *L2HGDH* gene expression (Spearman’s rank Rho = 0.55, Fig. 2B,E). In contrast, as shown in Fig. 2B,D, *HIF1A* CNV had a weak correlation with its own gene expression (Spearman’s rank Rho = 0.28), indicating that the preserved allele compensated for the genetic loss in 14q deleted kidney cancer. Genetic loss of *HIF1A* was therefore associated with a much greater reduction of *L2HGDH* gene expression than its own gene expression (Fig. 3A), providing a possible explanation for survival differences based on *HIF1A* CNV and mRNA expression. For comparison within the same dataset, survival based on *L2HGDH* expression has been shown in Fig. 3B (lower *L2HGDH* expression is associated with markedly worse survival in ccRCC). Furthermore, there was no correlation between *HIF1A* mRNA and HIF1α protein expression (Pearson’s correlation R = 0.02) (Fig. 2F).

### The small minority of 14q-deleted ccRCC patients in whom *L2HGDH* is not involved (but *HIF1A* is) has greater survival than the large majority in whom both genes are involved

In ccRCC patients with complete (uncensored) survival data (TCGA), patients with “*HIF1A* shallow deletion + *L2HGDH* diploid” (n = 5) had significantly greater mean survival (1670.8 days; 95% CI 1311.1–2030.5, n = 5) compared to patients with “*HIF1A* shallow deletion + *L2HGDH* shallow deletion” (885.1 days, 95% CI 798.9–971.3, n = 87) (t-test p value = 0.007) (Fig. 3C). Kaplan Meier survival curves of the two groups showing a wide separation between the curves are depicted in Fig. 3D (Peto and Peto modification of the Gehan-Wilcoxon test ‘p’ value = 0.033). Put together, when genetic loss of *HIF1A* occurs without genetic loss of *L2HGDH* in ccRCC, the survival is significantly greater than when there is simultaneous genetic loss of *HIF1A* and *L2HGDH*, which argues against *HIF1A* being a target of 14q deletion, and against *HIF1A* being a 14q tumor-suppressor in ccRCC.

The low sample size (n = 5) of 14q-deleted ccRCC patients with complete survival data having “*HIF1A* shallow deletion + *L2HGDH* diploid” is due to the relative rarity of this occurrence. As such, the survival data presented in Fig. 3C,D would need to be further validated in larger datasets when available. However, despite the low sample size, the magnitude of difference in survival was large enough to meet statistical significance.

### 14q has several yet-to-be-characterized potential tumor suppressors in ccRCC, even between the loci of *L2HGDH* and *HIF1A*

Of the 76 total protein-coding genes between *L2HGDH* at 14q21.3 and *HIF1A* at 14q23.2 (also deleted in ≥ 95% of 14q deletions in ccRCC involving *HIF1A*), there were 26 genes whose lower expression was significantly associated with inferior survival (log-rank p < 0.05), and are yet to be functionally characterized in ccRCC. These 26 genes are presented in Table 1 (including location; hazard ratio for higher expression vs lower expression separated by median; CNV and gene expression correlation) as well as Fig. S1. Of these 26 genes, 14 were also under-expressed in ccRCC compared to normal kidney tissue, and all these 14 genes had a stronger correlation between their respective copy numbers and gene expression than *HIF1A*, indicating that their preserved alleles did not compensate for the genetic loss as much as the preserved allele for *HIF1A* in 14q deleted RCC (mean CNV- expression correlation for the 14 genes was 0.51, compared with 0.27 for *HIF1A*).

**Table 1:** List of 26 genes between the loci of *L2HGDH* at 14q21.3 and *HIF1A* at 14q23.1, whose lower gene expression is significantly associated with inferior survival in ccRCC (logrank p < 0.05) but are yet to be functionally characterized.

Gene	Location	Hazard ratio# (high vs low expression)	CNV/mRNA correlation	Under-expressed in ccRCC tumor vs normal kindey?## (% reduction in expression log_2_(TPM + 1) in ccRCC vs normal)
***CDKL1*** (L-2-Hydroxyglutarate Dehydrogenase)	14q21.3	0.5	0.31	Yes (32.7%)
***ATL1*** (Atlastin GTPase 1)	14q22.1	0.54	0.2	No
***SAV1*** (salvador family WW domain containing protein 1)	14q22.1	0.55	0.66	Yes (12.8%)
***NIN*** (ninein)	14q22.1	0.55	0.15	No
***TMX1*** (thioredoxin related transmembrane protein 1)	14q22.1	0.52	0.45	No
***FRMD6*** (FERM domain containing 6)	14q22.1	0.55	0.34	No
***TXNDC16*** (thioredoxin domain containing 16)	14q22.1	0.53	0.44	No
***GPR137C*** (G protein-coupled receptor 137C)	14q22.1	0.52	0.35	No
***FERMT2*** (fermitin family member 2)	14q22.1	0.59	0.49	Yes (13%)
***CNIH1*** (cornichon family AMPA receptor auxiliary protein 1)	14q22.2	0.57	0.55	No
***GMFB*** (glia maturation factor beta)	14q22.2	0.55	0.44	Yes (14.3%)
***CGRRF1*** (cell growth regulator with ring finger domain 1)	14q22.2	0.55	0.58	Yes (13.6%)
***FBXO34 ***(F-box protein 34)	14q22.3	0.43	0.62	Yes (17.1%)
***ATG14*** (autophagy related 14)	14q22.3	0.67	0.59	No
***KTN1*** (kinectin 1)	14q22.3	0.49	0.52	Yes (12.1%)
***PELI2*** (pellino E3 ubiquitin protein ligase family member 2)	14q22.3	0.43	0.38	Yes (37.9%)
***EXOC5*** (exocyst complex component 5)	14q22.3	0.52	0.5	No
***AP5M1*** (adaptor related protein complex 5 subunit mu 1)	14q22.3	0.4	0.51	Yes (22.9%)
***NAA30*** (N(alpha)-acetyltransferase 30, NatC catalytic subunit)	14q22.3	0.55	0.62	Yes (14.3%)
***ACTR10*** (actin related protein 10)	14q23.1	0.58	0.67	Yes (6.7%)
***ARID4A*** (AT-rich interaction domain 4A)	14q23.1	0.6	0.46	No
***KIAA0586***	14q23.1	0.58	0.54	No
***DAAM1 ***(dishevelled associated activator of morphogenesis 1)	14q23.1	0.52	0.44	Yes (28.9%)
***JKAMP ***(JNK1/MAPK8 associated membrane protein)	14q23.1	0.54	0.59	No
***PPM1A*** (protein phosphatase, Mg2 + /Mn2 + dependent 1A)	14q23.1	0.44	0.66	Yes (14.3%)
***TRMT5 ***(tRNA methyltransferase 5)	14q23.1	0.56	0.68	Yes (13.8%)

These genes are also deleted in ≥ 95% of ccRCC 14q deletions involving the *HIF1A* locus. The location; hazard ratio for higher expression vs lower expression separated by median; CNV and gene expression correlation are presented (Also see Fig. S1). Of the 26 genes, 14 were also under-expressed in ccRCC compared to normal kidney (Also see Fig. S2), and all these 14 genes also had a stronger correlation between their respective copy number and gene expression than *HIF1A*, indicating that their preserved alleles did not compensate for the genetic loss compared to that of *HIF1A* in 14q- deleted ccRCC.

^#^Hazard ratio for overall survival based on higher vs lower expression of specified gene, separated by median.

^*##*^Under-expressed defined as > 5% reduction in log_2_(TPM + 1) in ccRCC vs normal kidney (TCGA).

Given the combination of lower expression in ccRCC tumor compared to normal kidney, lower expression associated with worse survival in ccRCC, and a strong CNV-gene expression correlation, these 14 genes display characteristics of potential 14q tumor suppressor genes in ccRCC. It is possible that some of these genes are not functionally tumor suppressive, and that their association with survival is secondary to a strong correlation between their gene expression and that of *L2HGDH* (along with other co-deleted functionally tumor suppressive genes).

Of all the 28 genes (including *HIF1A* and *LHGDH*), *L2HGDH* had the greatest percentage reduction in expression in clear cell renal cancer compared to normal kidney (40% reduction in log_2_(TPM + 1)), followed by *PELI2* (38%) and *CDKL1* (33%) (TCGA, Fig. S2A–C). Furthermore, survival effect size comparison between the genes revealed that only one of the 28 genes (*AP5M1*) had a hazard ratio (HR) for higher vs lower expression separated by median (0.4) lower than that of *L2HGDH* (0.42), although several other genes in the list had HR values between 0.43 and 0.6 (Fig. S1). These data further highlight the prominent tumor-suppressive role of *L2HGDH in* this malignancy.

*HIF1A* was among the higher expressed genes both in normal kidney and clear cell renal cancer (TCGA, Fig. S2A–C). While this may imply that the gene *HIF1A* is transcriptionally more active than most other genes in the list (or that its expressed transcript has lesser inhibitory regulation), it does not by itself account for the preserved-allele compensation of *HIF1A* being high in 14q-deleted ccRCC. *KTN1,* despite being expressed as high as *HIF1A,* has much lower preserved allele compensation, and a CNV/mRNA correlation of 0.52, nearly two times that of *HIF1A* (0.27). Analysis of promoter methylation of genes with high preserved-allele compensation and low CNV/mRNA correlation (*HIF1A* and *ATL1*) compared with those having low preserved-allele compensation and high CNV/mRNA correlation (*TRMT5* and *ACTR10*) did not reveal biologically significant differences in mean methylation beta values (Fig. S3). Even the least expressed of the genes listed in Table 1, *GPR137C*, had similarly low promoter methylation beta values (Figs. S2, S3). Future studies including chromosome conformation capture in ccRCC tumors (including 14q-deleted ccRCC) may reveal the mechanistic basis for preserved-allele compensation of *HIF1A*.

## Discussion

Clear cell Renal Cell Carcinoma (ccRCC) is the only malignancy in which HIF1α has been stated to have a tumor-suppressive role. The claim was primarily based on proliferation studies in ccRCC cell lines and their xenograft models in nude mice with HIF1α manipulation^35^. While the data were compelling, they stood in contrast with the well-established tumor-promoting properties of HIF1α in other malignancies (via angiogenesis, metabolic adaptation, resistance, etc.). The paper further stated that loss of *HIF1A* (which is located at 14q23.2, and encodes HIF1α) was a target of 14q loss in kidney cancer (14q deletion is seen in up to 40% of ccRCC). Following this landmark paper, the poor prognosis of 14q deleted RCC patients was commonly attributed to loss of *HIF1A*^39^. Although this claim was questioned^41^, from a translational standpoint, inhibiting HIF1 as an anti-cancer strategy has since been viewed as murky at best, and likely harmful in kidney cancer.

Recently, HIF-2 inhibition- resistant ccRCC patient-derived xenograft tumors were found to have high *HIF1A* levels, raising the question whether HIF1α indeed played a tumor promoting/resistance-conferring role in these ccRCC tumors^43^. Then, within the last two years, the tumor suppressive role of L2HGDH (L-2-Hydroxyglutarate Dehydrogenase) was characterized in ccRCC-based on mechanistic in vitro and in vivo studies^45^ as well as patient histology and survival data^44^. The characterization of L2HGDH as a tumor suppressor was much more comprehensive than HIF1α.

This article reports that *L2HGDH* is located reasonably close to *HIF1A* on 14q (at 14q21.3), and is deleted in ~ 95% of 14q deletions in ccRCC involving the *HIF1A* locus. Furthermore, the copy number of *HIF1A* correlates much stronger with *L2HGDH* expression (Rho = 0.55) than its own gene expression (Rho = 0.27) revealing that there is a high degree of preserved-allele compensation of *HIF1A* in 14q deleted RCC (compared to *L2HGDH* as well as other potential 14q tumor-suppressors identified in this article). Most importantly, HIF1α data from this large cohort of ccRCC patients (TCGA) at the mRNA (n = 530) and protein (n = 444) levels showed that it has no impact on survival, arguing against the claim that HIF1α functions as a tumor suppressor in the only malignancy in which it has been claimed to have this role. Despite the weak positive correlation between *HIF1A* and *L2HGDH* expression, the survival curves for cases with higher mRNA/higher protein expression/higher activity (CA9 expression) trended slightly lower than that of cases with lower mRNA/lower protein expression/lower activity (CA9 expression). Furthermore, there is no correlation between *HIF1A* mRNA and HIF1α protein expression in ccRCC. Lastly, in 14q- deleted ccRCC patients with complete (uncensored) survival data, in the relatively rare cases where genetic loss of *HIF1A* occurs without genetic loss of *L2HGDH*, the survival is significantly greater than when there is simultaneous genetic loss of *HIF1A* and *L2HGDH*. Taken together, the data presented in this study indicate that HIF1α likely has tumor promoting activity in ccRCC (similar to other malignancies), or less likely has a neutral role in terms of tumor progression, and certainly not a tumor suppressive role in ccRCC patients. As an extension, it can be reasonably concluded that the poor prognosis of 14q-deleted ccRCC is not due to genetic loss of *HIF1A*.

The regulation of HIF1α is primarily post-translational; as a result, even though the *HIF1A* transcript is lesser than that of normal kidney tissue (13.4% reduction in log2(TPM + 1) units in tumor vs normal, TCGA KIRC), HIF1α protein expression has been consistently shown to be higher than normal kidney^28,31,32^, a feature not consistent with a tumor suppressor. It is possible that HIF1α plays a more important oncogenic role in ccRCC pathogenesis and early progression, than later in the progression of advanced disease.

This study by no means questions the published experimental observations with in vitro and in vivo studies demonstrating a reduction in cancer cell proliferation with *HIF1A* over-expression or increase in cancer cell proliferation with *HIF1A* knockdown, or the mechanistic studies showing HIF1α induced inhibition of c-myc^29,34–38^. However, it does question the characterization and labeling of HIF1α as a ‘tumor-suppressor’ in kidney cancer based on experimental observations in models with no angiogenesis (in-vitro cell lines) or with deficient immune system (nude mice xenograft). The actions of some proteins/pathways within cancer cells involve a high degree of interaction with the cancer microenvironment as a part of their cumulative role. HIF1α is one such protein. Angiogenesis is a well-known important function of the HIFs. Furthermore, HIF1α within cancer cells promotes myeloid derived suppressor cells (MDSCs) in the tumor microenvironment^49^. Nude mice lack T cells and therefore do not fully capture the immunosuppressive (and tumor-promoting) effect of MDSCs. For these reasons, the usefulness of in-vitro cell lines and nude mouse models to determine the comprehensive role of HIF1α in human ccRCC tumors is limited, particularly in light of ccRCC patients’ tissue expression/survival data providing evidence to the contrary. This study also refutes the labeling of HIF1α as a target of 14q deletion in kidney cancer, and the claim that the poor prognosis of 14q-deleted ccRCC patients is due to genetic loss of *HIF1A* (by revealing a high degree of *HIF1A* preserved-allele compensation and its relation with recently characterized ccRCC tumor suppressor *L2HGDH* in 14q deletions).

The immunocompetent syngeneic mouse RCC model (RENCA) overcomes the limitations of the above models, with a major caveat, however, being that it is a mouse RCC, not human. Interestingly, in this model, HIF1α mediates VHL loss induced enhancement of epithelial mesenchymal transition and increased migratory/invasive capacity^42^. The VHL knockout RENCA cells demonstrated increased migratory/invasive capacity, reversed by H1F1A knockout. However, the same VHL knockout RENCA cells had reduced proliferation in vitro when compared to control, and the same primary tumor weight as control after orthotopic injection (despite the fact that VHL is a well-known tumor-suppressor). In addition, consistent with the observation of HIF1α enhancing invasion/migration in RENCA, microarray analysis of ccRCC tumors classified as ‘H1H2’ (expressing both HIF1α and HIF2α) had a significantly stronger ‘growth factor signaling/motility’ signature than ccRCC tumors classified as ‘H2′ (expressing HIF2α, not HIF1α) (Fig. 3D in^29^).

Furthermore, recent experimental observations in our lab were consistent with the reduced proliferation with VHL knockout reported in RENCA^42^**.** Pre-cancerous kidney cells (HK-2) with CRISPR mediated mono-allelic VHL knockdown proliferated much slower than the wild type cells in culture flasks; and over time, the normal allele compensated for the VHL protein loss (Fig. S4), associated with improved proliferation. We were unable to generate HK-2 colonies with a bi-allelic VHL knockout despite multiple attempts. In keeping with the inability to generate biallelic VHL knockout clones in the HK-2 cell line, Dr. Tien Hsu, corresponding author of^50^, also stated that HK-2 clones with stable VHL knockdown could not be maintained for long (personal communication). Does this now mean that VHL functions as a ‘tumor-promoter’ given the proliferation experimental observations in RENCA and HK-2 cell lines?

Taken together, relying only on ‘proliferation’ data in vitro and in vivo with the currently available pre-clinical models is not adequate to determine the comprehensive tumor suppressive/promoting effects of HIF1α (or HIF-VHL pathway) in human cancer.

In the future, technological advance may allow us to capture the expression and activity of a protein in real time during the various stages of cancer progression. Until then, however, we have two model systems: **A.** far-from- accurately-representative preclinical models for mechanistic studies (particularly so for some proteins/pathways), and **B.** large human cancer datasets with clinical and molecular annotations (which represent human disease but do not allow for mechanistic studies). In order for a protein to be branded a ‘tumor-suppressor’ in a particular human malignancy, satisfying the ‘tumor-suppressor’ criteria of model system B (i.e., lower expression/activity associated with worse pathologic features and survival, and reduced expression in cancer compared to normal tissue in general) is as important as satisfying the criteria of model system A (with genetic manipulation in-vitro and in-vivo). This is particularly true for a protein such as HIF1α, which has a profound influence on human cancer microenvironment (rendering model system A poorly representative of human disease), and has discordant results in the two model systems. (As an analogy, when a drug is considered to be a promising anti-cancer agent based on in vitro and in vivo models but then fails to demonstrate any anti-tumor effect in humans, it is no longer considered an effective anti-cancer agent).

Unlike HIF1α, L2HGDH has satisfied the criteria for both model systems in ccRCC. Its loss results in accumulation of the oncometabolite L2HG, which in turn inhibits the TET enzymes leading to genome-wide hypermethylation and reduced expression of multiple tumor-suppressors. L2HGDH has been shown to suppress in vitro cell migration and in vivo tumor growth. Its expression is lower in ccRCC compared to normal kidney tissue, and lower tumoral expression is associated with markedly worse survival in ccRCC patients^44,45^. Furthermore, this article shows that 14q loss in ccRCC results in much greater reduction in *L2HGDH* levels than *HIF1A* levels, indicating much lower preserved-allele compensation of *L2HGDH* than *HIF1A*. Lastly, when genetic loss of *HIF1A* occurs without genetic loss of *L2HGDH* in ccRCC, the survival is significantly greater than when there is simultaneous genetic loss of *HIF1A* and *L2HGDH.* These data, as well as the comparative expression and survival analyses presented in this article (Table 1 and Figs. S1–S3) make *L2HGDH* a far better candidate for a “target of 14q deletion”. However, labeling one particular gene as a “target” of large chromosomal deletions severely discounts the potential impact of several other co-deleted potential tumor suppressor genes. Table 1 and Fig. S1 of this article further emphasize this point. The impact of regulatory elements lost with the large deletion also merits theoretical consideration.

The robustness of the TCPA RPPA data used in this study may be considered a potential limitation despite the large sample size (given the concern that the technique can be error-prone). However, the RPPA data for ccRCC shows expected survival difference based on known general tumor-promoters (such as c-myc, P-cadherin, etc.) and tumor-suppressors (such as PTEN, Tuberin, etc.), arguing for its validity/robustness. The rationale for including RPPA (both HIF1α and CAIX) in this article was to highlight the survival difference based on protein expression/activity within the same dataset as that for RNA-based expression/survival analyses. Furthermore, the study with the largest sample size investigating the immunohistochemical expression of HIF1α and its relation with survival in ccRCC (n = 308) showed that ccRCC had much higher HIF1α IHC expression than normal kidney, and also that higher expression was associated with significantly worse survival (p = 0.005), arguing against HIF1α being a tumor-suppressor in the disease^28^, in accordance with the RPPA data. Another limitation in this study is the small number (n = 5) of ccRCC patients with uncensored survival data with 14q deletions affecting the *HIF1A* locus but not the *L2HGDH* locus. The low sample size is due to the relative rarity of this occurrence. The survival data presented in Fig. 3C,D would therefore need to be further validated in larger datasets when available. However, despite the low sample size, the magnitude of difference in survival was large enough to meet statistical significance. Lastly, while the survival analyses based on gene expression were not controlled for factors such as age or tumor stage, the large sample size of the study minimizes its effect on overall interpretation and conclusions.

Cell lines and their xenograft models in rodents do hold an important place in cancer research. However, they capture only a fraction of actual tumor biology of human cancers, and do not account for much of the interactions between cancer cells and the tumor microenvironment^51^. As such, there is a need to recognize and accept the limitations of our pre-clinical models in representing human cancer pathophysiology in certain contexts, such as defining the comprehensive tumor-suppressive/promoting role of proteins with a marked influence on the cancer microenvironment. The limitations are even more evident today, with the availability of large human cancer datasets with extensive molecular/clinical annotations that enable analyses such as those presented in this article.

From a future translational standpoint, combinatorial targeting of HIF2α and HIF1α may have a role in ccRCC treatment (perhaps particularly in a subset with high HIF1α protein expression/activity), and deserves exploration.

## Conclusion

Taken together, the data indicate that HIF1α is not a target of 14q deletion in clear cell renal cancer and that it is not a tumor-suppressor in this malignancy.

Furthermore, while L2HGDH is a far better candidate for a “target of 14q deletion”, labeling one gene as a “target” of large chromosomal deletions severely discounts the impact of several other co-deleted potential tumor suppressor genes that are yet to be characterized.

## Materials and methods

Bioinformatic analysis of the TCGA and TCPA Kidney Clear Cell Carcinoma (KIRC) was performed using multiple integrative web-based platforms.

**The University of California Santa Cruz Xena Platform**^52^ for cancer genomics data visualization and interpretation was used to analyze and generate Figs. 1A,B, 3B. The TCGA KIRC registry was selected and interrogated for *HIF1A* CNV, *HIF1A* mRNA and L2HGDH mRNA (n = 530). Kaplan Meier Survival analysis was then performed based on *HIF1A* CNV/mRNA expression (Fig. 1A,B) and *L2HGDH* mRNA expression (Fig. 3B). The Xena platform was also used to interrogate and generate CNV/mRNA correlations for the 26 genes listed in Table 1, and data for Fig. S2A,B.

**The Gene Expression Profiling Interactive Analysis (GEPIA)**^53^ platform was used to further confirm and validate the survival data (ensuring that the survival curves generated with the **Xena**^52^ platform were accurate and reproducible). The GEPIA platform was also used to interrogate and generate Fig. S1 (A–Z) hazard ratios presented in Table 1, and data for Fig. S2C.

**The Cancer Proteome Atlas (TCPA)** web-based platform^54^ was used to analyze and generate Fig. 1C,D. The TCGA KIRC registry was selected and interrogated for survival based on HIF1α protein expression (n = 444) and CA9 protein expression (n = 444).

**Heatmapper.org** was used to generate Fig. 2A, with pre-clustered TCGA clear cell RCC data for *HIF1A* CNV and mRNA, *L2HGDH* CNV and mRNA (Raw data downloaded from TCGA via the **Xena**^52^ platform was clustered in Excel and entered in **Heatmapper.org** to generate the heatmap).

The **cBioPortal**^55^ platform was used to analyze and generate Fig. 3A. The TCGA KIRC registry was selected and interrogated for *L2HGDH* and *HIF1A* expression with ‘diploid’ or ‘shallow deletion’ of *HIF1A* locus (among ccRCC patients with available mutation and copy number alteration data). P-value was calculated with 2-sided t-test in Excel after extracting the raw data from cBioPortal.

**Atlasgeneticsoncology.org** was used to identify genes between the loci of *L2HGDH* and *HIF1A*. These genes were individually analyzed with the **Xena**^52^ platform (TCGA KIRC and TCGA normal kidney registries) to generate data presented in columns 3 and 4 of Table 1 (as mentioned above).

**Microsoft Excel** was used to analyze and generate Fig. 2B–E, with TCGA/TCPA clear cell RCC raw data downloaded via the **Xena**^52^ and **LinkedOmics**^56^ platforms. Figure S2A–C were also generated in Microsoft Excel, with relevant TCGA KIRC and normal kidney tissue data downloaded from Xena^52^ and GEPIA^53^.

**Survival analysis based on copy number alterations of HIF1A and L2HGDH** (Fig. 3C,D): All ccRCC TCGA patients with available copy number alteration data on *HIF1A* and *L2HGDH* (n = 509) were downloaded from cbioportal. The KIRC clinical data was then downloaded from the Firebrowse platform. The copy number data was then matched to ‘days to death’ data in excel using the ‘vlookup’ function. Patients with complete (uncensored) survival data were included for analysis to ensure robustness (given the small number of patients in the ‘HIF1A shallow deletion/L2HGDH diploid’ group and high likelihood of mismatch in censoring credibility between the two arms). There were 89 patients in the “*HIF1A* shallow deletion/*L2HGDH* shallow deletion” group (group 1) and 6 patients with uncensored survival data in the “*HIF1A* shallow deletion/*L2HGDH* diploid” group (group 2). The 89 patients in group 1 had the following survival (days) from diagnosis: 1191, 137, 1610, 885, 480, 683, 139, 106, 182, 600, 1337, 202, 168, 1588, 1019, 183, 307, 2386, 1238, 834, 1639, 793, 883, 1121, 2145, 637, 362, 222, 459, 333, 68, 1598, 927, 480, 1003, 709, 992, 1912, 1493, 344, 1270, 162, 480, 334, 1097, 62, 1912, 822, 2227, 431, 1661, 336, 646, 3554, 1567, 819, 841, 1173, 139, 572, 2190, 782, 574, 679, 768, 1620, 3615, 2299, 224, 1964, 164, 41, 571, 1404, 2105, 206, 446, 2830, 561, 330, 311, 2564, 73, 59, 946, 445, 1626, 242. The 6 patients in group 2 had the following survival (days) from diagnosis: 2241, 18, 1724, 1584, 1714, 1091. The median survival 768 days for group 1, and 1649 days for group 2. Therefore, among patients with complete (uncensored) data, the median survival in “HIF1A shallow deletion/L2HGDH diploid” group was 2.15 times the median survival of “*HIF1A* shallow deletion/*L2HGDH* shallow deletion” group. For calculation of the mean survival difference between the groups (Fig. 3C), given that outliers significantly affect the means, the standard outlier test (any number less than Q1 – 1.5 × IQR or greater than Q3 + 1.5 × IQR, IQR being Q3–Q1) was applied to both groups. ‘3615’ and ‘3554’ were outliers in group 1, and ‘18′ was an outlier in group 2. Student’s t-test was used to calculate the statistical significance of difference between the two groups given that only complete (uncensored) data were used for analysis. Kaplan Meier survival curves for the 2 groups depicted in Fig. 3D.

**TCGA Wanderer**^57^ platform was use to perform methylation analysis and generate figures for the five genes analyzed and presented in Fig. S3.

**USC Genome Browser**^58^ was used to generate Fig. 2A, depicting the location of *L2HGDH* in relation to that of *HIF1A* on chromosome 14q.

## Supplementary information

Supplementary information 1

Supplementary information 2
