# Supplementary material for: HIF1α is not a target of 14q deletion in clear cell renal cancer
Source: Sci Rep. 2020 Oct 19;10:17642. doi: 10.1038/s41598-020-74631-7 (PMC7573601; doi:10.1038/s41598-020-74631-7)
Supplement: Supplementary file 1 — Supplementary information 1 [file 41598_2020_74631_MOESM1_ESM.pdf]

HIF1α is not a target of 14q deletion in clear cell renal cancer

Niraj Shenoy, MD, PhD, MS [1,2]

1 Department of Medicine (Oncology), Albert Einstein College of Medicine, Montefiore Medical Center, New York 10461
2 Experimental Therapeutics Program, Albert Einstein Cancer Center, Albert Einstein College of Medicine, New York 10461

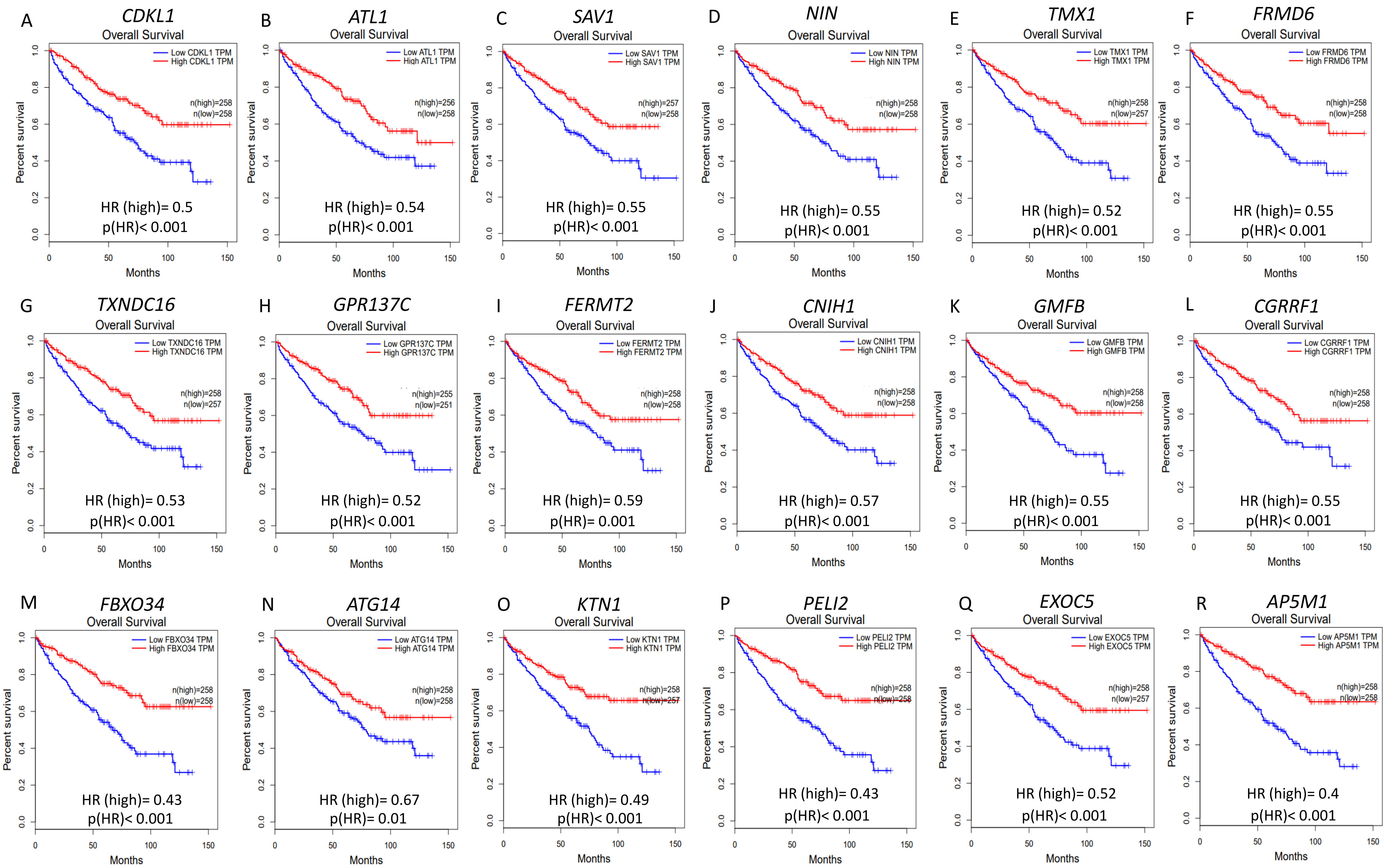
A CDKL1 Overall Survival
Low CDKL1 TPM
High CDKL1 TPM
n(high)=258
n(low)=258
HR (high)= 0.5
p(HR)< 0.001
B ATL1 Overall Survival
Low ATL1 TPM
High ATL1 TPM
n(high)=256
n(low)=258
HR (high)= 0.54
p(HR)< 0.001
C SAV1 Overall Survival
Low SAV1 TPM
High SAV1 TPM
n(high)=257
n(low)=258
HR (high)= 0.55
p(HR)< 0.001
D NIN Overall Survival
Low NIN TPM
High NIN TPM
n(high)=258
n(low)=258
HR (high)= 0.55
p(HR)< 0.001
E TMX1 Overall Survival
Low TMX1 TPM
High TMX1 TPM
n(high)=258
n(low)=257
HR (high)= 0.52
p(HR)< 0.001
F FRMD6 Overall Survival
Low FRMD6 TPM
High FRMD6 TPM
n(high)=258
n(low)=258
HR (high)= 0.55
p(HR)< 0.001
G TXNDC16 Overall Survival
Low TXNDC16 TPM
High TXNDC16 TPM
n(high)=258
n(low)=257
HR (high)= 0.53
p(HR)< 0.001
H GPR137C Overall Survival
Low GPR137C TPM
High GPR137C TPM
n(high)=255
n(low)=251
HR (high)= 0.52
p(HR)< 0.001
I FERMT2 Overall Survival
Low FERMT2 TPM
High FERMT2 TPM
n(high)=258
n(low)=258
HR (high)= 0.59
p(HR)= 0.001
J CNIH1 Overall Survival
Low CNIH1 TPM
High CNIH1 TPM
n(high)=258
n(low)=258
HR (high)= 0.57
p(HR)< 0.001
K GMFB Overall Survival
Low GMFB TPM
High GMFB TPM
n(high)=258
n(low)=258
HR (high)= 0.55
p(HR)< 0.001
L CGRRF1 Overall Survival
Low CGRRF1 TPM
High CGRRF1 TPM
n(high)=258
n(low)=258
HR (high)= 0.55
p(HR)< 0.001
M FBXO34 Overall Survival
Low FBXO34 TPM
High FBXO34 TPM
n(high)=258
n(low)=258
HR (high)= 0.43
p(HR)< 0.001
N ATG14 Overall Survival
Low ATG14 TPM
High ATG14 TPM
n(high)=258
n(low)=258
HR (high)= 0.67
p(HR)= 0.01
O KTN1 Overall Survival
Low KTN1 TPM
High KTN1 TPM
n(high)=258
n(low)=257
HR (high)= 0.49
p(HR)< 0.001
P PELI2 Overall Survival
Low PELI2 TPM
High PELI2 TPM
n(high)=258
n(low)=258
HR (high)= 0.43
p(HR)< 0.001
Q EXOC5 Overall Survival
Low EXOC5 TPM
High EXOC5 TPM
n(high)=258
n(low)=257
HR (high)= 0.52
p(HR)< 0.001
R AP5M1 Overall Survival
Low AP5M1 TPM
High AP5M1 TPM
n(high)=258
n(low)=258
HR (high)= 0.4
p(HR)< 0.001
Percent survival
Months

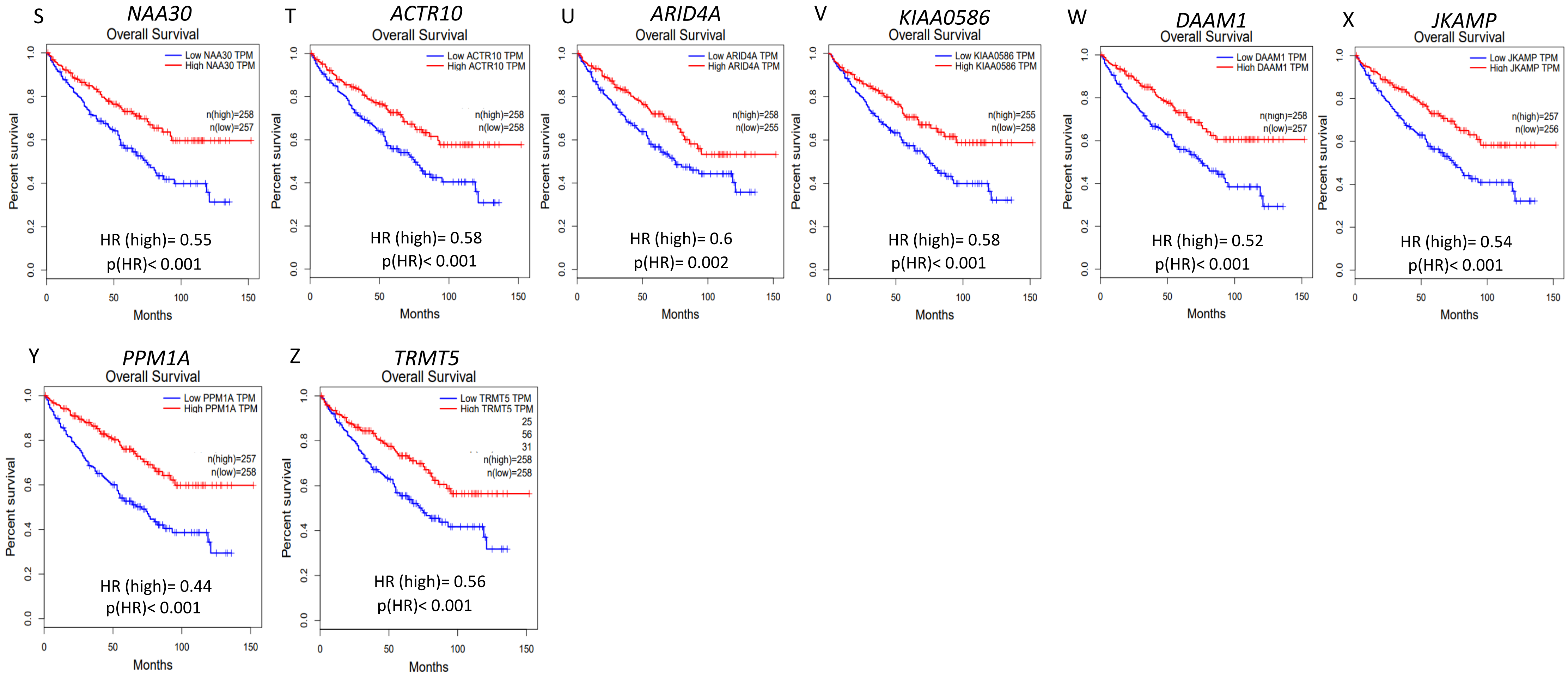

Figure S1. A–Z: 26 genes between the loci of *L2HGDH* at 14q21.3 and *HIF1A* at 14q23.1, whose lower gene expression is significantly associated with inferior survival in ccRCC (log-rank $p < 0.05$) but are yet to be functionally characterized. The figures show survival curves based on higher or lower expression of the gene, separated by median (TCGA). Hazard ratios are depicted, as well as the p values for hazard ratio. These 26 genes are also deleted in ≥ 95% of ccRCC 14q deletions involving the *HIF1A* locus. (*Of note, for comparison of effect size, HR (high) value for L2HGDH, a fully characterized 14q ccRCC tumor suppressor, is 0.42. Only one of the 26 genes (AP5M1) has a HR (high) value lower than that of L2HGDH)*

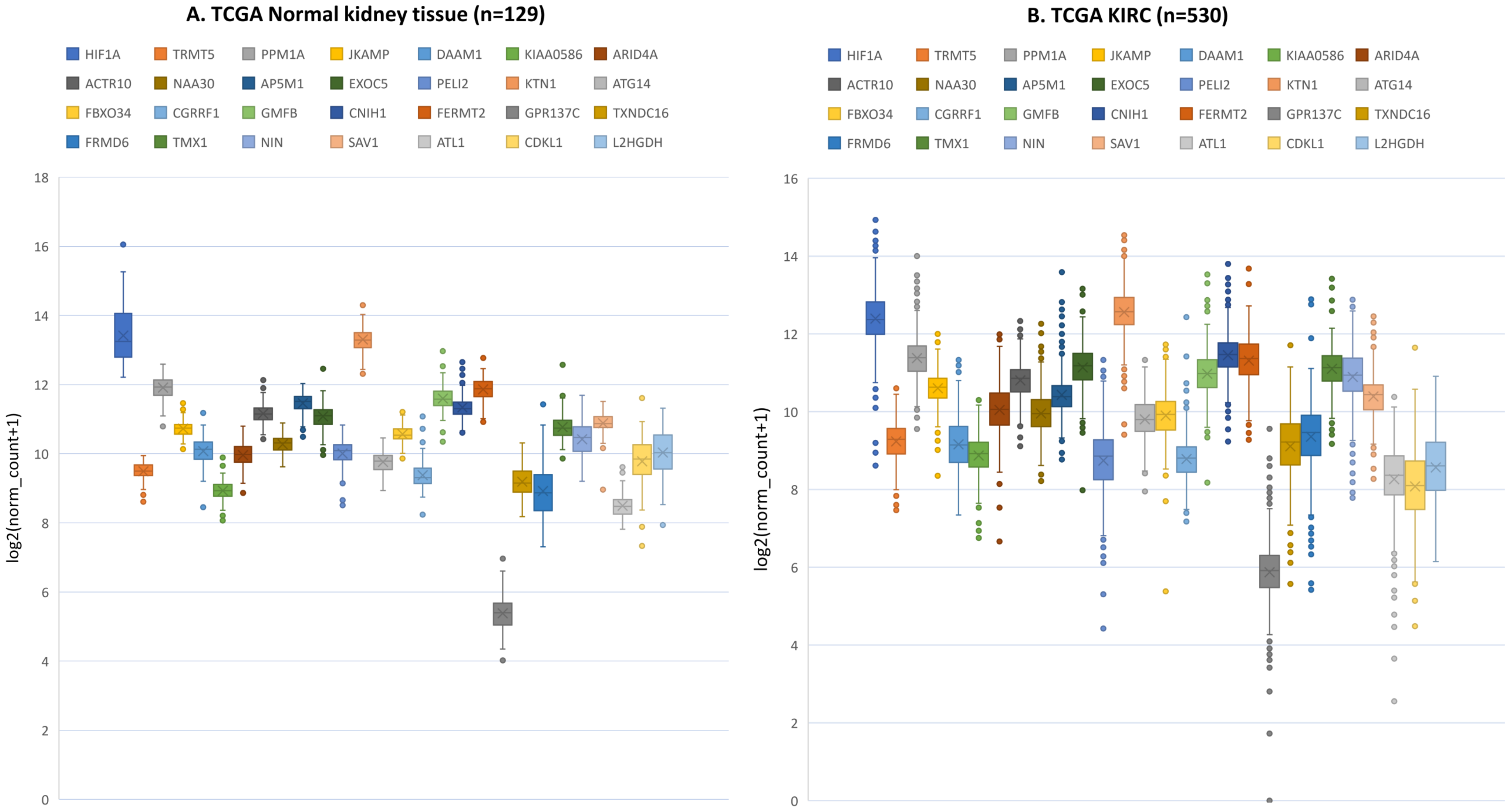

Figures S2A,B. Expression of the 26 genes depicted in Table 1 in log2 (norm_count+1) units (along with the two genes *HIF1A* and *L2HGDH*). TCGA normal kidney tissue (**S2A**, n=129) and TCGA KIRC (**S2B**, n=530).

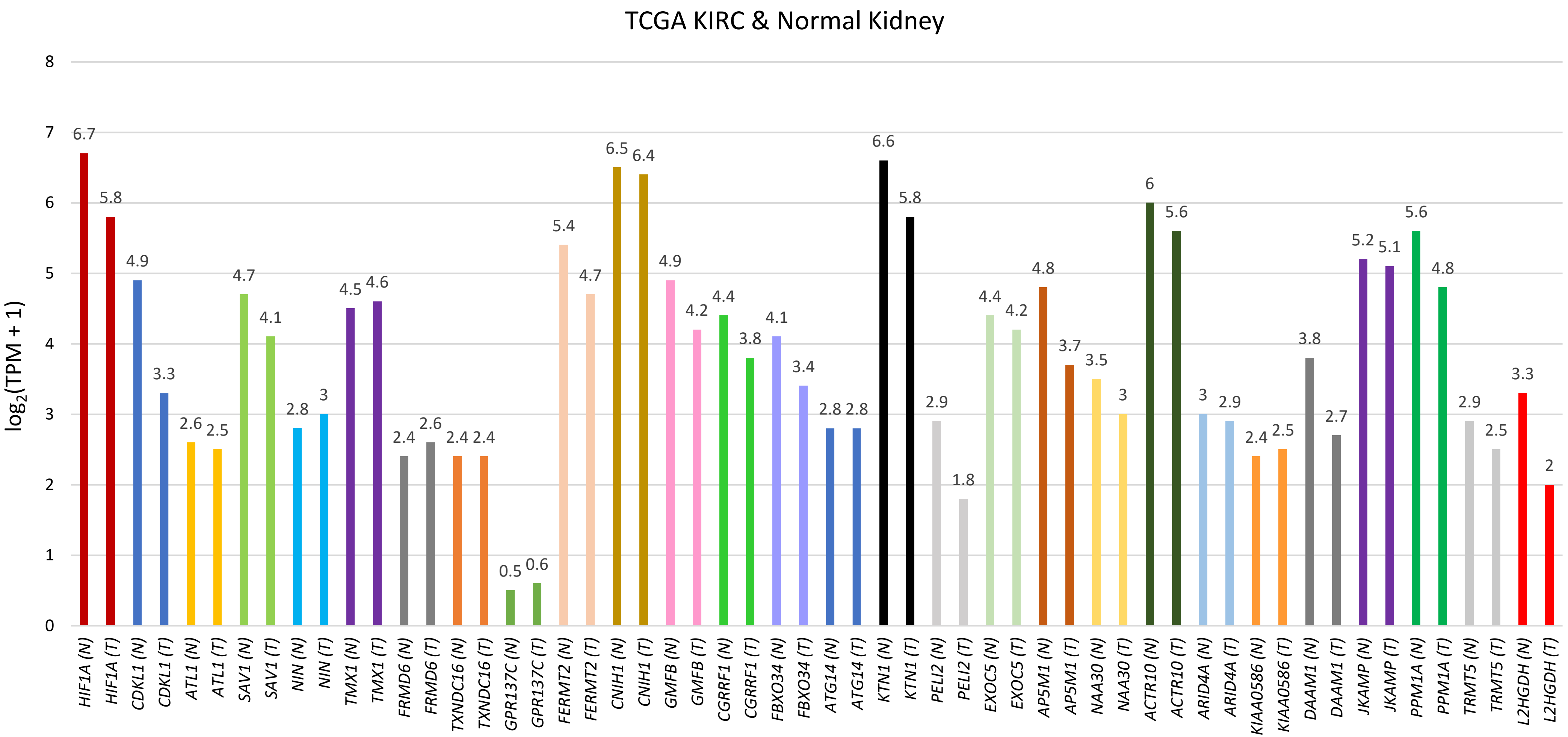

Figure S2C. Expression of the 28 genes in TCGA KIRC and normal kidney in log2 (TPM+1) units to facilitate comparison of expression between genes. Of all the 28 genes, *L2HGDH* (far right) has the greatest percentage reduction in expression in tumor compared to normal (40% reduction), followed by *PELI2* (38%) and *CDKL1* (33%). *HIF1A* is among the higher expressed genes, both in tumor and normal tissue.

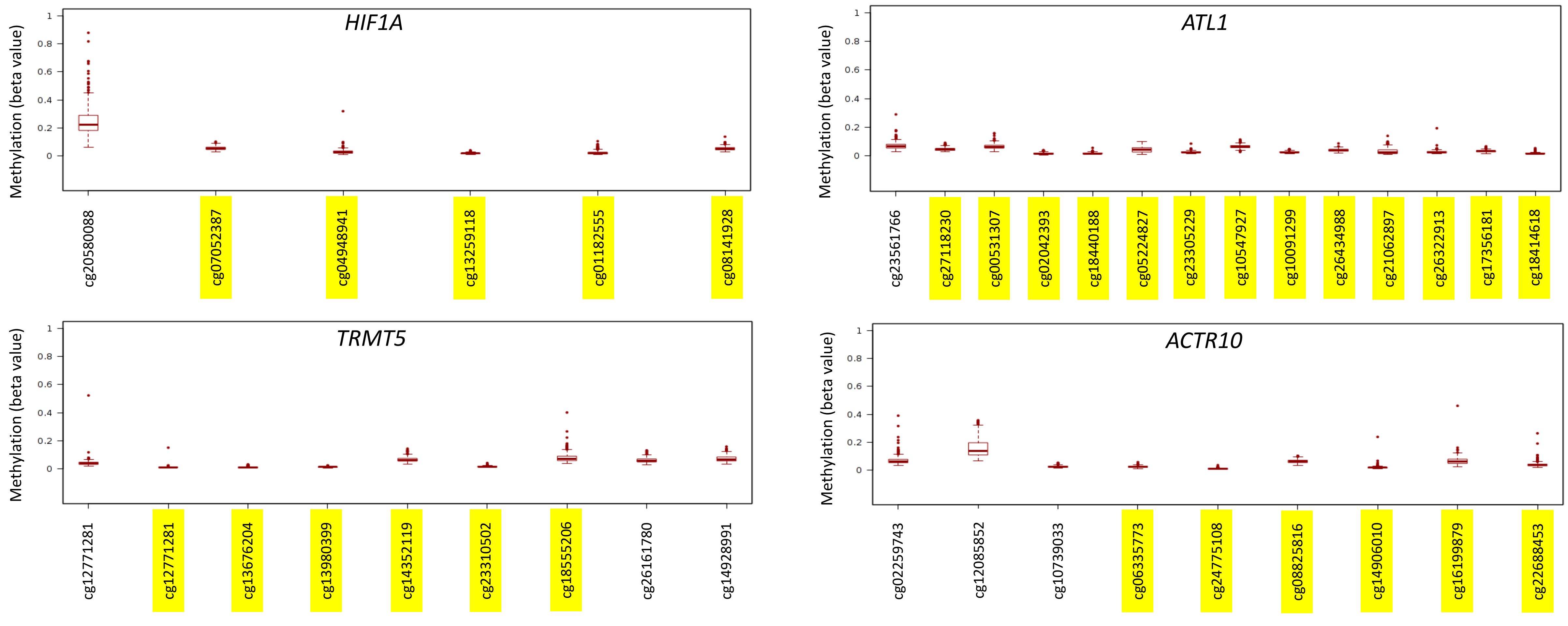

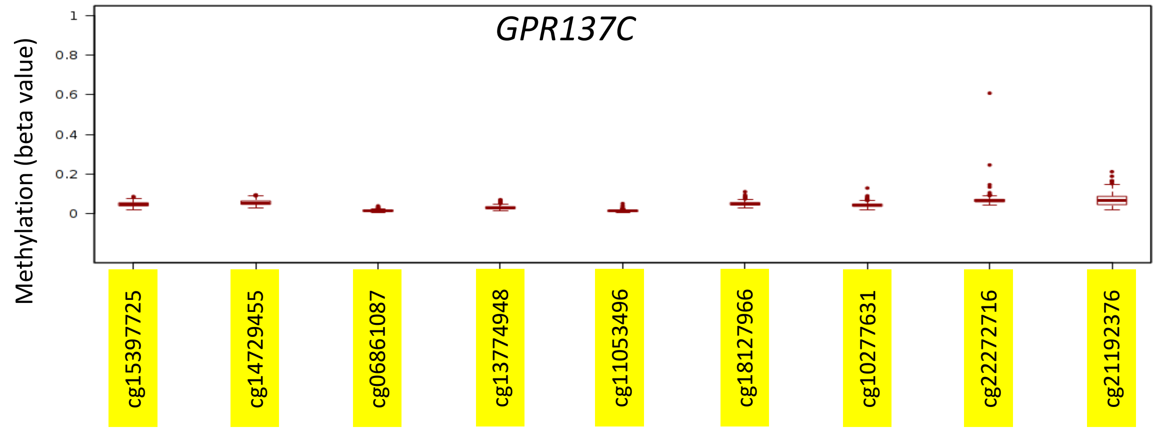

Figure S3. CpG island probes are highlighted. Non- CpG island probes are not. Probes for 1000bp upstream and 500 bp downstream from TSS of each gene depicted. Low methylation beta values seen for all the 5 analyzed genes, including *HIF1A*. TCGA KIRC (n= 324).

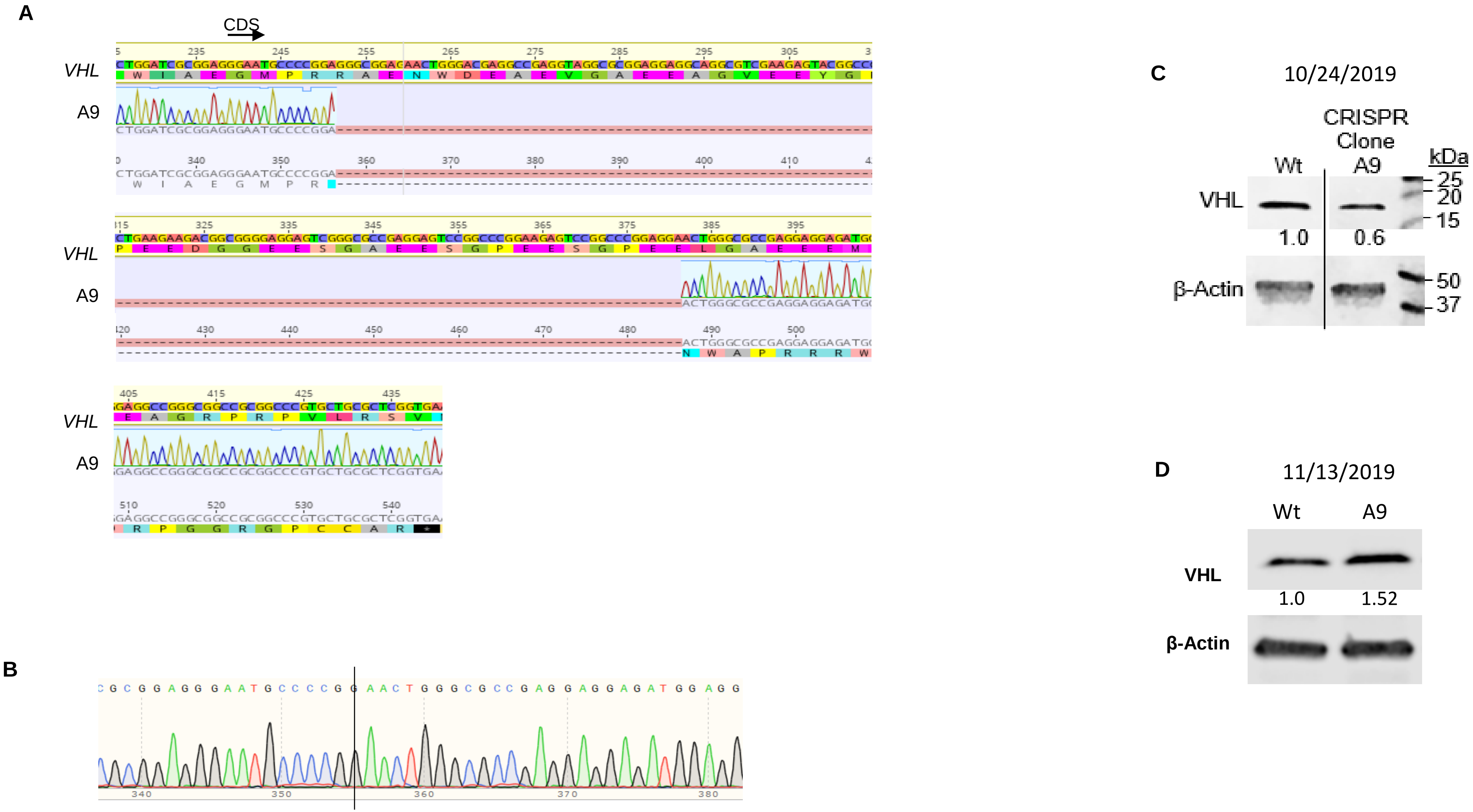

Figure S4. A. Alignment to VHL genomic sequence shows 130 bp deletion in Exon 1 resulting in a frame-shift and introduction of an early stop codon. **B.** Sanger sequence of deleted region. **C, D.** A9 clone western blot done 3 weeks apart, revealing increased VHL expression (compensation by the preserved allele) over time. Figure S4C represents non-contiguous lanes from a single blot. *(The A9 clone was generated as a part of another project in the lab; this particular observation turned out relevant for the discussion section of this manuscript)*
