# Supplementary material for: HIF1α is not a target of 14q deletion in clear cell renal cancer
Source: Sci Rep. 2020 Oct 19;10:17642. doi: 10.1038/s41598-020-74631-7 (PMC7573601; doi:10.1038/s41598-020-74631-7)
Supplement: Supplementary file 2 — Supplementary information 2 [file 41598_2020_74631_MOESM2_ESM.pdf]

HIF1α is not a target of 14q deletion in clear cell renal cancer

Niraj Shenoy, MD, PhD, MS [1,2]

1. Department of Medicine (Oncology), Albert Einstein College of Medicine, Montefiore Medical Center, New York 10461
2. Experimental Therapeutics Program, Albert Einstein Cancer Center, Albert Einstein College of Medicine, New York 10461

10.24.19

kDa HK2 lines: Wt A9

50
37
25
20
15

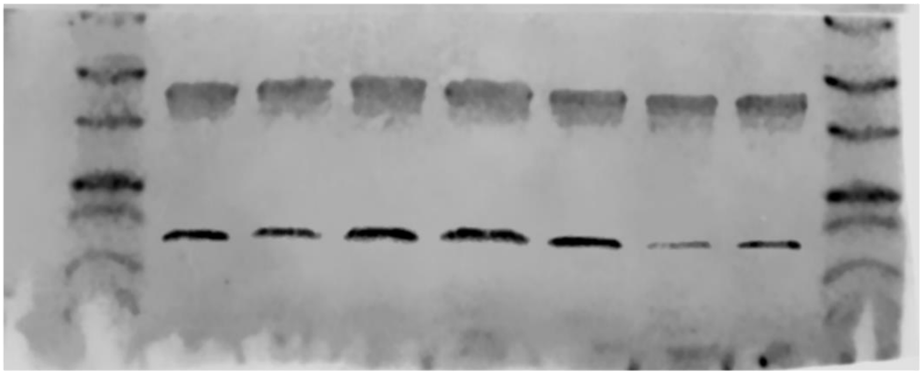

β-Actin

VHL

11.13.19

kDa HK2 lines: Wt A9

50
37
25
20
15

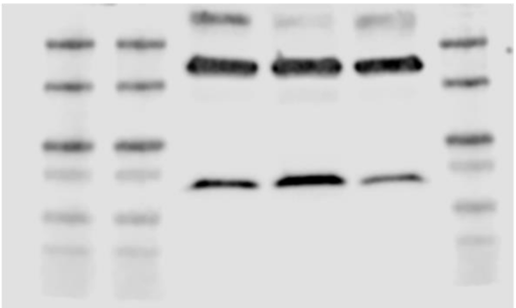

β-Actin

VHL

Full-length blots for figures S4C, S4D
